# Supplementary material for: Development of chemokine network inhibitors using combinatorial saturation mutagenesis
Source: Commun Biol. 2025 Apr 3;8:549. doi: 10.1038/s42003-025-07778-6 (PMC11969024; doi:10.1038/s42003-025-07778-6)
Supplement: Supplementary file 4 — Supplementary Data 2 [file 42003_2025_7778_MOESM4_ESM.zip › SupplementaryData3/scripts/xattune_ic50.tar!/./script/analysis/JK/JK_R26_E63_RA_HD2CM307_ATC_plot.pdf]

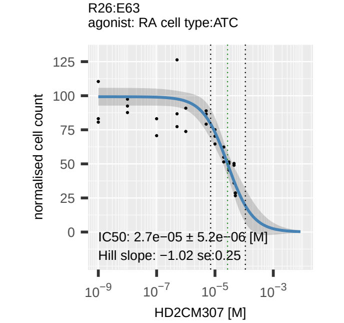

R26:E63
agonist: RA cell type:ATC
IC50: 2.7e−05 ± 5.2e−06 [M]
Hill slope: −1.02 se:0.25
normalised cell count
125
100
75
50
25
0
10−9
10−7
10−5
10−3
HD2CM307 [M]
